# ARDS Subphenotypes: Understanding a Heterogeneous Syndrome

**DOI:** 10.1186/s13054-020-2778-x

**Published:** 2020-03-24

**Authors:** Jennifer G. Wilson, Carolyn S. Calfee

**Affiliations:** 1 Department of Emergency Medicine, Stanford University, Palo Alto, CA USA; 2 Department of Medicine, University of California, San Francisco, San Francisco, CA USA; 3 Department of Anesthesia, University of California, San Francisco, San Francisco, CA USA

## Abstract

This article is one of ten reviews selected from the Annual Update in Intensive Care and Emergency Medicine 2020. Other selected articles can be found online at https://www.biomedcentral.com/collections/annualupdate2020. Further information about the Annual Update in Intensive Care and Emergency Medicine is available from http://www.springer.com/series/8901.

## Introduction

The acute respiratory distress syndrome (ARDS) is a clinical syndrome defined by acute onset hypoxemia (PaO_2_:FiO_2_ ratio < 300) and bilateral pulmonary opacities not fully explained by cardiac failure or volume overload [1]. The Berlin consensus definition of ARDS, like the American-European Consensus definition that preceded it, has enabled clinicians and researchers alike to prospectively identify patients with ARDS, implement lung protective ventilation strategies, and enroll patients in clinical trials. ARDS remains under-recognized clinically, however; therapies are limited, and mortality remains high [2]. Under-recognition of ARDS may stem in part from the considerable clinical heterogeneity observed among patients who meet standard ARDS criteria. The syndrome may be triggered, for example, by pulmonary or extrapulmonary sepsis, aspiration, trauma, blood product transfusion, or pancreatitis. Pulmonary infiltrates can be focal or diffuse. Hypoxemia can range from mild to severe, and duration of respiratory failure can be brief or prolonged. Many of these clinical variations may reflect underlying biological differences between ARDS patients that are now recognized as important drivers of treatment response and ultimate outcomes.

Substantial heterogeneity within the general ARDS population has likely contributed to the failure of experimental therapies for ARDS in recent large clinical trials, despite promising preclinical data [3]. Identifying subphenotypes of ARDS— more homogeneous groups within the general ARDS population—is one approach to untangling the clinical and biological complexity that many believe is a barrier to discovery of successful new treatments. By identifying meaningful but currently unrecognized subgroups encompassed by the broad consensus definition of ARDS, interventions can potentially be tested more efficiently in targeted cohorts. Selecting subphenotypes of patients at higher risk for poor outcomes for enrollment in clinical trials is called prognostic enrichment [4]. Selecting for patients more likely to respond to a given therapy due to the mechanism of benefit is called predictive enrichment [4]. Both enrichment strategies are recommended by the Food and Drug Administration to increase the efficiency of clinical trials across all fields, either by increasing the rate of the outcome of interest (prognostic enrichment) or by amplifying the effect size (predictive enrichment). These approaches may allow researchers to detect treatment effects in smaller cohorts, which is especially important in heterogeneous syndromes like ARDS. Ultimately, however, the discovery of ARDS subphenotypes may enrich more than clinical trial populations: within the next decade, these innovations could help us move from a one-size-fits-all approach to ARDS treatment to more effective, tailored therapies based on the clinical and biologic profile of each patient.

This chapter summarizes the state of the science of subphenotyping of ARDS patients, exploring the physiologic, clinical, and biologic characteristics that have been found to identify more homogeneous subgroups within this heterogeneous syndrome (Table 1), and the potential implications of these advances for practicing clinicians in the intensive care unit (ICU) and the emergency department.

**Table 1:** Examples of factors used for identifying subphenotypes of the acute respiratory distress syndrome (ARDS)

Physiologic	Clinical	Biologic
PaO_2_:FiO_2_	Trauma vs. medical	Genomic
Dead space fraction	Direct vs. indirect	Transcriptomic
Driving pressure	Focal vs. diffuse	Proteomic
	±Acute kidney injury	Metabolomic

### ARDS Subphenotypes and Prognostic Enrichment

Prognostic enrichment in ARDS research involves selecting patients with a higher likelihood of having a particular disease-related endpoint, such as fewer ventilator-free days or higher mortality. Beyond increasing research efficiency, identifying subphenotypes of ARDS patients at highest risk for poor outcomes may also lead to improved risk stratification at the bedside, allowing clinicians to select patients more likely to benefit from inter-facility transfer for higher level of care, or early consideration of aggressive therapies such as extracorporeal membrane oxygenation (ECMO).

### Physiologic Phenotyping for Prognostic Enrichment

Risk stratification of ARDS patients is not a new strategy. The Berlin definition itself stratifies ARDS into three subgroups (Table 2) according to the degree of hypoxemia (mild, moderate, and severe), and mortality increases as the PaO_2_:FiO_2_ ratio decreases [1]. The advantage of this approach is that the PaO_2_:FiO_2_ ratio is available in all patients with ARDS and does not require expert interpretation or subjective clinical assessment. Multiple large clinical ARDS trials have used the PaO_2_:FiO_2_ ratio for prognostic enrichment. For example, the ACURASYS trial of early continuous neuromuscular blockade [5], the PROSEVA trial of prone positioning [6], and the ROSE trial reevaluating early continuous neuromuscular blockade [7] all targeted patients with moderate-to-severe ARDS (PaO_2_:FiO_2_ ratio < 150 mmHg). All three of these trials had mortality endpoints, and all three had mortality rates in the control arms that exceeded 40%.

**Table 2:** The Berlin Definition of ARDS categorizes patients according to the severity of their oxygenation deficit; increasing severity is associated with increased mortality [1]

Severity	PaO_2_:FiO_2_ ratio (mmHg)	Patients (%)	Mortality (%)
Mild	201–300	22	27
Moderate	101–200	50	32
Severe	≤100	28	45

In addition to the PaO_2_:FiO_2_ ratio, several other physiologic variables are known to predict poor outcomes in ARDS. Dead space fraction [8], ventilatory ratio (a simple bedside index of impaired ventilation) [9], and driving pressure (a measurement of respiratory system compliance) are all independently associated with poor outcomes in ARDS [10] and more routine measurement of these variables could improve prognostic enrichment in clinical trials and risk prediction in clinical practice.

One limitation of using these physiologic measurements, however, is that these variables can rapidly change. The application of higher positive end-expiratory pressure (PEEP), for example, could rapidly move a patient from one subgroup of ARDS severity to another, or a patient who aspirates at the time of intubation may develop severe hypoxemia that improves within hours. Perhaps more fundamentally, common physiologic characteristics in most cases do not capture important differences in biology between ARDS patients. A patient with transfusion-associated ARDS may have the same PaO_2_:FiO_2_ ratio or driving pressure as a patient with ARDS from H1N1 influenza, but their underlying pathophysiology may be very different, and they do not have the same risk of poor outcomes. Indeed, the Berlin definition of ARDS is far from perfect as a predictor of mortality, with an area under the curve of only 0.577 [1].

### Clinical Phenotyping for Prognostic Enrichment

Recognizing the limitations of a purely physiologic approach to subphenotyping ARDS patients, investigators have also examined various clinical variables to enhance prognostic enrichment (Table 1). For example, patients with ARDS following trauma have been found to be at lower risk of death than non-trauma patients with ARDS (odds ratio 0.44) [11]. Luo et al. found that despite overall similar mortality rates, predictors of mortality differ between direct (pulmonary trigger) and indirect (extrapulmonary trigger) ARDS [12]. ARDS patients with acute kidney injury (AKI) have been shown to have significantly higher mortality than patients without AKI in several cohorts [13, 14]. Thus, when attempting to identify high-risk ARDS patients, non-trauma patients and patients with significant AKI are a higher-risk subset, but predictors of mortality differ depending on whether the lung injury is direct or indirect.

Beyond baseline clinical characteristics, the time-course of ARDS is another factor that can identify patients at greater risk of poor outcomes. Both time of onset and duration of disease appear to hold prognostic value. Several studies have shown that ARDS onset >48 h after ICU admission is associated with higher mortality [15, 16]. Not surprisingly, rapidly improving ARDS (ARDS that resolves within 1 day) has a better prognosis than persistent ARDS. More interesting, however, is the finding that most (63%) patients with rapidly improving ARDS present with moderate or severe hypoxemia [17], highlighting the limitations of using the PaO_2_:FiO_2_ ratio alone to identify patients for enrollment in clinical trials. Recognizing this issue, the PROSEVA trial of prone positioning only enrolled patients if they continued to meet inclusion criteria (PaO_2_:FiO_2_ ratio < 150 mmHg) after 12–24 h of stabilization [7].

Radiographic patterns of pulmonary infiltrates have also been used to sort ARDS patients into more homogeneous subgroups and identify those at highest risk of death, either alone or in combination with other physiologic and clinical variables. One small prospective study found that ARDS patients with non-focal infiltrates had higher mortality compared to patients with focal radiographic findings [18]. The CESAR trial of ECMO for severe ARDS required a Murray Lung Score (which incorporates chest radiography) of >3 for eligibility (or a pH < 7.20) [19, 20]. More recently, the RALE score—developed to systematically quantify the extent and density of alveolar infiltrates on chest radiograph—has been shown to predict 28-day mortality with an area under the curve of 0.82 [21].

An apparent drawback to relying exclusively on clinical characteristics for phenotyping, however, is the potential for misclassification. In a recent trial of mechanical ventilation personalized according to the presence of focal vs. diffuse infiltrates in ARDS (discussed further later), 21% of the radiographic subphenotypes assigned at the time of randomization were misclassified [22]. Similarly, investigators have found it difficult to classify patients as having direct or indirect ARDS, with 37% of cases deemed unclassifiable in one trial [23]. Pragmatic and reliable approaches to classification are needed to overcome the challenges inherent to clinical phenotyping.

### Biologic Phenotyping for Prognostic Enrichment

**Fig. 1:**
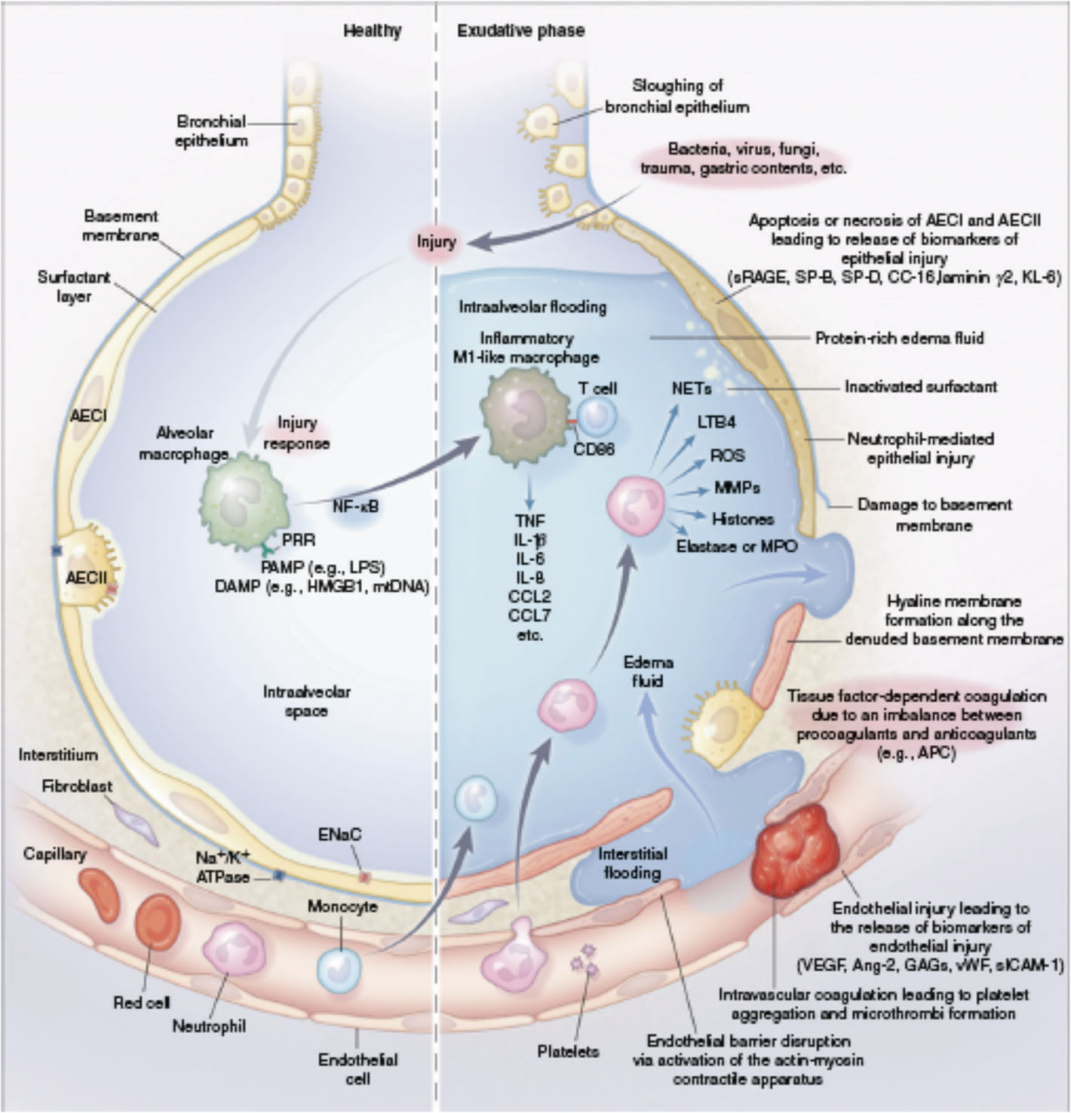
Pathobiology of the exudative phase of ARDS. The healthy alveolar-capillary unit (left) and the exudative phase of ARDS (right). *AECI* type I alveolar epithelial cell, *AECII* type II alveolar epithelial cell, *Ang-2* angiopoietin-2, *APC* activated protein C, *CC-16* club cell (formerly Clara cell) secretory protein 16, *CCL* chemokine (CC motif) ligand, *DAMP* damage-associated molecular pattern, *ENaC* epithelial sodium channel, *GAG* glycosaminoglycan, *HMGB1* high-mobility group box 1 protein, *KL-6* Krebs von den Lungen 6, *LPS* lipopolysaccharide, *LTB4* leukotriene B4, *MMP* matrix metalloproteinase, *MPO* myeloperoxidase, *mtDNA* mitochondrial DNA, *Na*^*+*^*/K*^*+*^ *ATPase* sodium-potassium ATPase pump, *NF-κB* nuclear factor kappa light-chain enhancer of activated B cells, *NET* neutrophil extracellular trap, *PAMP* pathogen-associated molecular pattern, *PRR* pattern recognition receptor, *ROS* reactive oxygen species, *sICAM* soluble intercellular adhesion molecule, *SP* surfactant protein, *sRAGE* soluble receptor for advanced glycation end products, *TNF* tumor necrosis factor, *VEGF* vascular endothelial growth factor, *vWF* von Willebrand factor. (Reused from [3] with permission)

It follows that there has been growing interest in identifying biologic subphenotypes of ARDS patients. Biologic markers are considered proximal to the clinical expression of ARDS, and potentially less prone to problems with misclassification that make clinical phenotyping so challenging. Moreover, our understanding of ARDS biology has advanced greatly in the past decade. We now better understand how an initial insult causes an inflammatory cascade that results in further injury to the alveolus and its microvasculature (Fig. 1) [3]. Measuring plasma biomarkers in ARDS can help find subgroups of patients that share important host-response features and/or that have worse clinical outcomes. Numerous genomic, transcriptomic, proteomic, and metabolomic factors have been studied for this purpose, with the greatest depth of research focused on plasma protein biomarkers of ARDS. These include markers of systemic inflammation (interleukin [IL]-6, IL-8, soluble tumor necrosis factor [TNF] receptor-1, IL-18), epithelial injury (angiopoietin-2, intercellular adhesion molecule-1), endothelial injury (soluble receptor for advanced glycation end products [sRAGE], surfactant protein-D), and disordered coagulation (plasminogen activator inhibitor-1, protein C), all of which have been shown to hold prognostic value [24]. Baseline levels of sRAGE, for example, independently predicted 90-day mortality in one meta-analysis [25]. More recently, Rogers et al. found that elevations in baseline plasma IL-18 levels and rising IL-18 levels were both associated with increased mortality in sepsis-induced ARDS [26].

**Fig. 2:**
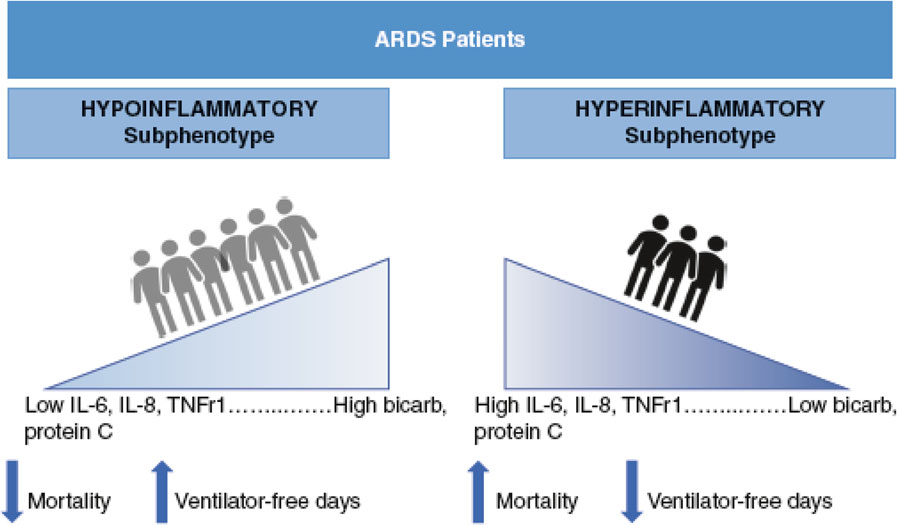
The hypoinflammatory and hyperinflammatory subphenotypes of ARDS are associated with different biomarkers and outcomes. These two distinct subphenotypes have been identified by Calfee et al. in multiple previous ARDS clinical trial cohorts [27, 29, 40, 41]. *IL* interleukin, *bicarb* bicarbonate, *TNFr1* tumor necrosis factor receptor 1

Using an approach to identify subgroups within a heterogeneous population called latent class analysis (LCA), two distinct subphenotypes of ARDS were identified based on combined clinical and biologic data from patients enrolled in two large clinical trial cohorts [27]. The “hyperinflammatory” subphenotype was characterized by enhanced inflammation, fewer ventilator-free days, and increased mortality compared to the “hypoinflammatory” subphenotype (Fig. 2). These two subphenotypes have been found in subsequent independent analyses of multiple other ARDS trial cohorts, and the poor prognosis associated with the hyperinflammatory phenotype persists [28, 29]. Using a different approach, called hierarchical clustering, to analyze a panel of plasma biomarkers from ARDS patients, Bos et al. identified two similar subphenotypes: a “reactive” subphenotype characterized by greater inflammation and increased mortality and an “uninflamed” subphenotype associated with better outcomes [30]. Taken together, these findings support the idea that ARDS patients can be stratified according to markers of inflammation for prognostic enrichment.

The focus on proteomic profiling of ARDS patients has been paralleled by interest in genomic, transcriptomic, and metabolomic subphenotypes of ARDS, but less progress has been made in terms of prognostic enrichment with these strategies. Meyer et al. identified an *IL-1RN* coding variant that increased risk of developing ARDS in sepsis [31], and Zhu et al. found certain micro-RNAs to be risk biomarkers for ARDS among critically ill adults [32], but genomic and transcriptomic subphenotyping of patients with established ARDS remains largely unexplored. In a small cohort of patients with ARDS, Rogers et al. found a subset of patients with a distinct metabolic profile with higher levels of numerous metabolites in undiluted pulmonary edema fluid [33]. This “high metabolite” subphenotype was associated with higher mortality but, in part because of the challenges associated with analyzing pulmonary edema fluid, these findings have yet to be reproduced in a larger cohort.

Indeed, measurement of protein biomarkers and “-omics” data in ARDS patients is not currently available outside of the research setting. Furthermore, the impact of identifying biologic subphenotype on downstream outcomes in ARDS patients has not been prospectively evaluated. Investigators have recently identified a “parsimonious” model that classifies ARDS patients as “hypo-” or “hyperinflammatory” using only three plasma biomarkers (IL-8, bicarbonate and protein C) [34], and rapid analysis of biomarkers for identification of ARDS subphenotype at the point of care is now being piloted. Development of rapid assays is a critical step in leveraging the identification of ARDS subphenotypes for prognostic enrichment in future clinical trials, and ultimately bringing these discoveries to the bedside.

### ARDS Subphenotypes and Predictive Enrichment

In parallel with these different strategies for identifying subgroups and phenotypes for prognostic enrichment in ARDS, investigators have also studied how treatment effects vary by subphenotype. By finding a subphenotype-specific treatment response retrospectively or targeting treatment based on mechanism and biologic features, researchers can then go on to prospectively test new interventions in patients who are more likely to respond. This approach provides predictive enrichment: by amplifying treatment response, the power to detect a benefit from experimental therapies increases, and discovery becomes more efficient. Just as biologic phenotyping of other diseases, such as breast cancer or asthma, has led to important improvements in patient outcomes, the eventual goal is to deploy targeted therapies for ARDS according to patient characteristics, moving the field from protocolized care to precision medicine.

### Physiologic Phenotyping for Predictive Enrichment

While many view physiologic parameters such as PaO_2_:FiO_2_ ratio as purely prognostic indicators, they may provide predictive enrichment as well. As Prescott et al. note in their discussion of ARDS clinical trial strategies, a lower PaO_2_:FiO_2_ ratio not only identifies patients at higher risk of death, but also reflects patients with greater lung weight who may be more likely to benefit from recruitment maneuvers, higher PEEP or prone positioning [35]. Similarly, one could hypothesize that among patients with severe ARDS, those who have a plateau pressure >30 cmH_2_O or an unfavorable driving pressure despite adherence to a lung-protective ventilation strategy may be more likely to benefit from “lung rest” with ultra-low tidal volumes on ECMO.

### Clinical Phenotyping for Predictive Enrichment

As discussed above, difficulty classifying patients is a pragmatic challenge inherent to clinical phenotyping of ARDS patients, and a major drawback to the use of clinical phenotyping in both research and practice. Nonetheless, different clinical subphenotypes of ARDS may reflect different underlying biology, and in some cases have been shown to respond differently to treatment. For example, patients with direct ARDS have higher levels of biomarkers of epithelial injury than patients with indirect ARDS [36], and there is low-level evidence that patients with direct ARDS may respond differently to recruitment maneuvers and glucocorticoids than indirect lung injury patients [22, 37–39]. A recent, highly innovative randomized controlled trial compared a personalized mechanical ventilation strategy selected according to radiographic subphenotype (focal vs. non-focal) to standard low tidal volume ventilation in 400 patients with moderate-to-severe ARDS (LIVE trial) [22]. The intention-to-treat analysis found no difference in outcomes between groups, but in a post hoc analysis that excluded misclassified patients (21% of total patients), there was a mortality benefit to the personalized mechanical ventilation intervention. These results highlight both the promise and the peril of using subphenotype (in this case radiographic subphenotype) to guide therapy: on the one hand, if patients are appropriately classified, there may be a benefit of personalized care over protocolized care; on the other hand, the significant misclassification that occurs even in the relatively controlled setting of a clinical trial can completely offset the potential benefit. Regardless of this complexity, the LIVE trial was a first step in the direction of what many view as the future of ARDS research: leveraging subphenotype to personalize therapy and directly comparing outcomes to standard protocolized care.

### Biologic Phenotyping for Predictive Enrichment

No trials have yet used biologic phenotyping pre-randomization, because bedside testing of biomarkers is not widely available. There is, however, mounting evidence that biologic phenotype predicts treatment response. In retrospective analyses, the hypo- and hyperinflammatory phenotypes discussed above have been observed to have differential treatment responses to several different interventions, including PEEP and fluid management strategies (Table 3) [27, 29]. Subphenotypic differences in response to simvastatin were also observed in reanalysis of one clinical trial (HARP-2 trial) [40], but not in a similar reanalysis of a separate trial of rosuvastatin for ARDS (SAILS trial) [41]. While it is possible this discrepancy reflects differences in trial design (and the particular statin that was tested), it also highlights the uncertainty that remains when differential treatment response has been observed only retrospectively.

**Table 3:** Subphenotype-specific treatment response in the reanalyses of outcomes in four different clinical ARDS trials

Intervention/trial cohort analyzed		Hypoinflammatory subphenotype response		Hyperinflammatory subphenotype response	
	Outcome	Intervention	Control	Intervention	Control
High vs. low PEEP/ ALVEOLI^*^ [27]	90-day mortality	24% high PEEP	16% low PEEP	42% high PEEP	51% low PEEP
Conservative vs. liberal fluid strategy/ FACCT^*^ [29]	90-day mortality	18% conservative fluid strategy	26% liberal fluid strategy	50% conservative fluid strategy	40% liberal fluid strategy
Simvastatin/ HARP-2 [40]	28-day survival	No difference		Improved survival with simvastatin (*p* = 0.008)	
Rosuvastatin/SAILS [41]	90-day mortality	No difference		No difference	

*PEEP* positive end-expiratory pressure; ^∗^*p* value <0.05 for interaction between treatment and subphenotype

The use of metabolomics, transcriptomics, and genomics for ARDS phenotyping and predictive enrichment is in even earlier stages than proteomic phenotyping. Bos et al. used the “uninflamed” and “reactive” subphenotypes they had previously identified based on plasma protein biomarkers to test whether there were differences in blood leukocyte gene expression between groups, and found that approximately one-third of genes were differentially expressed. Specifically, there was upregulation of oxidative phosphorylation genes in the “reactive” subphenotype, leading the authors to suggest that for patients in this group, interventions focused on this pathway should be explored [42]. While these data certainly merits further investigation, translating biologic associations into effective treatments based on mechanism is by no means straightforward. For example, a retrospective analysis of a previous negative clinical trial of recombinant IL-1 receptor antagonist for sepsis found a treatment benefit in the subset of patients with *higher* levels of baseline IL-1 receptor antagonist, arguably a completely counterintuitive result [43].

The benefit of biologically tailored precision therapies for ARDS remains theoretical. The translation of the insights gained from studying subphenotypes of ARDS into targeted therapies based on mechanism—and comparison of this precision approach to standard protocolized management—is the next frontier in ARDS research.

### Beyond ARDS: Subphenotypes in Other Heterogeneous Syndromes

As mentioned earlier, the search for subphenotypes in ARDS is motivated in part by improvements in the treatment of other heterogeneous diseases gained by using a similar approach. Oncologic therapies in particular are increasingly guided by molecular phenotype, and this strategy has improved survival substantially even in patients with advanced disease. Survival with metastatic melanoma, for example, has improved significantly since the advent of checkpoint inhibitors and therapies targeting the BRAF V600 mutation [44]. Asthma therapy has also been changed by the identification of clinically significant subphenotypes: patients with severe, uncontrolled eosinophilic asthma have been found to have fewer exacerbations with monoclonal antibodies aimed at reducing eosinophil activation [45].

Subphenotypes have also been identified in sepsis, another heterogeneous syndrome in the critically ill that has historically been treated with a protocolized approach. Wong et al. have developed a biomarker-based mortality risk model for pediatric sepsis, as well as gene-expression-based subphenotypes of pediatric septic shock. In a retrospective analysis of a cohort of pediatric patients with septic shock, they found that among intermediate- and high-risk patients, corticosteroids were associated with a more than tenfold reduction in the risk of a complicated course in one subphenotype but not the other [46]. Similar subphenotyping of adult sepsis is an area of active study. Gårdlund et al. used latent class analysis to identify six distinct subphenotypes of septic shock using clinical data from a previous large clinical trial cohort [47]. Seymour et al. used a different approach (machine learning applied to electronic health record data) and identified four subphenotypes with different genetic and inflammatory markers and markedly different mortality rates [48].

Finally, there is a small but growing body of evidence that there are meaningful subphenotypes within the heterogeneous post-cardiac arrest syndrome, beyond type of arrest and initial post-resuscitation neurologic status. For example, Bro-Jeppesen et al. have reported that IL-6, a marker of systemic inflammation, is correlated with poor prognosis in comatose patients resuscitated from out-of-hospital cardiac arrest [49]. Anderson et al. found that patients with post-resuscitation shock who had a preserved left ventricular ejection fraction (LVEF) had less favorable neurologic outcomes, increased organ failure, and higher mortality compared to patients with depressed LVEF [50]. In addition, patients in the preserved LVEF group exhibited a subtype-specific response to early fluid resuscitation, with lower mortality and improved neurologic outcomes associated with larger volume fluid resuscitation that was not observed in the group with depressed LVEF. These findings suggest that there are identifiable subphenotypes in post-cardiac arrest syndrome, and that subphenotypes may be important drivers of variable treatment response.

## Conclusion

The armamentarium of therapies for patients with ARDS remains limited and mortality remains high. While negative results in several large randomized controlled trials of new treatments for ARDS have frustrated many, they have also motivated multiple novel approaches to understanding the clinical and biologic heterogeneity among ARDS patients. The identification of meaningful ARDS subphenotypes—and the ways in which their outcomes and treatment responses differ—promises prognostic and predictive enrichment for future trials. Prospective evaluation of methods for reliable phenotyping at the point of care is a crucial next step in translating these discoveries into new personalized therapies for ARDS. Ultimately, identification of ARDS subphenotypes may help fulfill the aspiration of precision critical care for ARDS: replacing blunt interventions aimed at all patients who meet diagnostic criteria with therapies tailored to the clinical and biologic profile of each patient.

## Data Availability

Not applicable.
